# Early Changes in Costameric and Mitochondrial Protein Expression with Unloading Are Muscle Specific

**DOI:** 10.1155/2014/519310

**Published:** 2014-09-16

**Authors:** Martin Flück, Ruowei Li, Paola Valdivieso, Richard M. Linnehan, Josiane Castells, Per Tesch, Thomas Gustafsson

**Affiliations:** ^1^Balgrist University Hospital, University of Zurich, Switzerland; ^2^Laboratory for Muscle Plasticity, Balgrist University Hospital, Forchstrasse 340, 8008 Zurich, Switzerland; ^3^Institute for Biomedical Research into Human Movement and Health, Manchester Metropolitan University, Manchester, UK; ^4^National Aeronautics and Space Administration, Johnson Space Center, Houston, TX, USA; ^5^Laboratoire de Physiologie de l'Exercice, EA4338, Faculté de Médecine, Université Jean-Monnet, Saint Etienne Cedex, France; ^6^Department of Health Sciences, Mid Sweden University, Östersund, Sweden; ^7^Department of Physiology and Pharmacology, Karolinska Institute, Stockholm, Sweden; ^8^Department of Laboratory Medicine, Karolinska University Hospital, Clinical Physiology Karolinska Institute, Stockholm, Sweden

## Abstract

We hypothesised that load-sensitive expression of costameric proteins, which hold the sarcomere in place and position the mitochondria, contributes to the early adaptations of antigravity muscle to unloading and would depend on muscle fibre composition and chymotrypsin activity of the proteasome. Biopsies were obtained from vastus lateralis (VL) and soleus (SOL) muscles of eight men before and after 3 days of unilateral lower limb suspension (ULLS) and subjected to fibre typing and measures for costameric (FAK and FRNK), mitochondrial (NDUFA9, SDHA, UQCRC1, UCP3, and ATP5A1), and MHCI protein and RNA content. Mean cross-sectional area (MCSA) of types I and II muscle fibres in VL and type I fibres in SOL demonstrated a trend for a reduction after ULLS (0.05 ≤ *P* < 0.10). FAK phosphorylation at tyrosine 397 showed a 20% reduction in VL muscle (*P* = 0.029). SOL muscle demonstrated a specific reduction in UCP3 content (−23%; *P* = 0.012). Muscle-specific effects of ULLS were identified for linear relationships between measured proteins, chymotrypsin activity and fibre MCSA. The molecular modifications in costamere turnover and energy homoeostasis identify that aspects of atrophy and fibre transformation are detectable at the protein level in weight-bearing muscles within 3 days of unloading.

## 1. Introduction

The maintenance of skeletal muscle mass calls for continued mechanical stress [1]. This load dependence is illustrated by the decrease in size or volume of antigravity muscles after unloading induced by real or simulated microgravity [2, 3]. The consequences of unloading are evident at the molecular level in human skeletal muscle within 3 days [4, 5]. Reductions in the MCSA of muscle fibres are detectable within 4-5 days of unloading with immobilization [6, 7]. In rodents, atrophy occurs concurrently with a shift toward faster fibre types [8, 9]. Evidence suggests that the reduced energy expenditure associated with chronic unloading is accompanied by compromised aerobic capacity and mitochondrial function [10–13]; however, at the level of mitochondrial proteins in muscle, this may be masked by a pronounced muscle atrophy [14].

It is generally accepted that both reduced protein synthesis and increased degradation of myofibrillar proteins contribute to the skeletal muscle dysfunction that is caused by the lack of weight-bearing activity [5, 15–17]. This view is supported by the upregulation of transcripts that control the activity of proteasomal degradation in antigravity muscles after 2–4 days of ULLS and/or immobilization [4, 6, 18, 19]. This is in contrast with the situation observed after ULLS, in which the posttranslational regulation of prominent upstream regulators of protein translation (AKT, TSC-2, p70S6K, 4EBP1, eIF4E, and eEF2) is not affected after 3–10 days of unloading, despite a pronounced reduction in myofibrillar protein synthesis rate and muscle atrophy [4, 15, 17]. Only the tyrosine phosphorylation of the integrin-associated focal adhesion kinase (FAK), which acts upstream of ribosome biogenesis, was reduced after 10 days of ULLS [17, 20], and the level of the transcript for an inhibitor of protein synthesis (4E-BP1) was increased [4]. Collaterally, messenger RNAs for factors of mitochondrial metabolism are pronouncedly downregulated after 2 days of unloading in men [7, 18], which is consistent with the reported rapid decrease in oxidative enzyme activities and increased UCP3 content in rat fast muscle with unloading [13, 14]. Collectively, the reported adaptations support the view that disuse atrophy resulting from muscle unloading involves early regulation of gene expression towards an enhanced capacity of the proteasomal pathway, concomitant with a downregulation of protein synthetic capacity and mitochondrial biogenesis.

Regarding the coordination of the load-regulated mechanisms that maintain muscle mass, it is of relevance to consider the role of focal adhesions in the sarcolemma (costameres) [21]. Costameres assemble through the binding of cytoskeletal and signalling molecules at the intracellular side of integrin-type and/or dystrophin/sarcoglycan-type extracellular matrix receptors [22–24]. They serve as attachment sites for the intermediate filaments that hold myofibrils in place and position mitochondria respective to myofibrils [24, 25], thereby enabling sarcomerogenesis [26]. Costameres also integrate mechanical cues into the activation of biochemical signalling toward gene expression and protein translation and cytoskeletal organization of striated muscle (reviewed in [23, 27]). Importantly, both reduced and increased muscle loading modify costamere-component content, and these alterations correlate with changes in muscle thickness [27–29]. Changes in the concentration of the costamere components, paxillin and gamma- and metavinculin, can be detected as early as 7 days after unloading in rodent skeletal muscle (reviewed in [27, 30, 31]). These changes correspond to a sizable reduction in tyrosine 397 phosphorylation (pY397) of FAK, which also regulates costamere-component expression and turnover in a load-dependent fashion [27, 29]. The current knowledge indicates that modified costamere-component expression and posttranslational modification of FAK [24, 32, 33] represent early events in muscle remodelling.

A striking observation regarding muscle remodelling by atrophy caused by unloading is its muscle-type dependence [34, 35]. This resembles the alterations in the level of costamere components observed in both rats and humans with reduced load bearing (reviewed in [27, 30, 31]). For instance, metavinculin, paxillin, and FAK were downregulated in the (mixed) fast-type plantaris and gastrocnemius muscles (reviewed in [30, 31]), whereas metavinculin level increased with unloading in the slow oxidative soleus (SOL) muscle (reviewed in [30, 31]). In rodents, this mode of regulation relates to the differential modulation of tyrosine phosphorylation of FAK and of the expression of its endogenous inhibitor, the FAK-related nonkinase (FRNK) [27], in slow-type compared with fast-type muscles [31]; moreover, it has been shown to involve the mechanoregulation of the slow oxidative gene programme [20]. These observations suggest that changes in the expression profile of costameric factors and downstream transcript expression may represent adaptive processes and the phenotype of a mechanically challenged muscle.

In the current study, we investigated whether 3 days of unloading initiated fibre transformation at the protein level, as reflected in altered expression of use/load-sensitive costameric and mitochondrial proteins in the VL and SOL muscles. A 3-day intervention was selected to evaluate the early effects of unloading on the expression of costamere components, which are related to mechanosensory muscle remodelling via the organization of the attachment of muscle organelles and gene expression [24–27]. We further speculated that these responses would differ between the phenotypically distinct leg VL and SOL muscles and would reflect differences in the activity of chymotrypsin, a main contributor to the 20S proteasome, being increased within 5 days of unloading in the rat [36], and/or expression of costameric proteins. Subsequently, we assessed whether quantitative, linear relationships existed between unloading-induced changes in chymotrypsin activity and fibre MCSA and between the levels of selected proteins that define the slow oxidative muscle phenotype and whose expression is subject to regulation by FAK protein and its pY397 content [20, 37].

## 2. Materials and Methods

### 2.1. Subjects

Healthy men (mean ± SD; 25 ± 5 yr, 183 ± 3 cm, 76 ± 8 kg, *n* = 8) were recruited from the metropolitan area of Stockholm. The subject sample ranged from physically active to sedentary individuals. Subjects were screened for any history of lower limb pathology, neuromuscular disorder, or cardiovascular disease. Written consent was obtained from each subject after being informed of the procedures, risks, and potential benefits associated with the experiments. The study protocol was approved by the Ethics Committee at the Karolinska Institutet and conducted in accordance with the Declaration of Helsinki (1964).

### 2.2. Unloading

Unilateral limb suspension (ULLS) was accomplished as described [4, 5]. In brief, upright or ambulatory activities were aided by short-length crutches with handgrip and forearm support distal to the elbow (Swereco Rehab AB, Sollentuna, Sweden). The right foot was equipped with a shoe outfitted with a 10 cm thick sole in order to remove weight-bearing load from the left unloaded limb. There were no straps attached to the shoe restraining ankle- or knee-joint movement. The subjects lived at home and maintained their normal occupational tasks throughout the experimental period. Compliance was encouraged through daily interaction with one of the investigators. Two weeks prior to the ULLS intervention, all subjects underwent four sessions to practice walking on crutches and daily tasks associated with the ULLS intervention. The subjects refrained from any strenuous physical activity three days prior to the onset of ULLS yet maintained their normal dietary habits. To assure compliance, all subjects were interviewed daily via telephone or in person.

### 2.3. Collection of Muscle Samples

Muscle biopsies were obtained from the right leg prior to ULLS and from the left leg after completing 72 hrs of ULLS and before resuming any weight-bearing activity. Following an overnight fast and after injection of local anaesthetic (Carbocaine) and skin incision, biopsies were obtained from VL and SOL using a 5 mm Bergström needle. Samples were cleansed of excess blood, connective tissue, and fat and then frozen in liquid nitrogen and stored at −80°C until further analysis.

### 2.4. Immunoblotting

Muscle biopsies were cross-sectioned at 25 micrometers using a cryostat; proteins were isolated with rotor stator mixer (Ultraturrax, IKA Werke GmbH & Co. KG, Germany) into ice-cold modified radio immunoprecipitation buffer (RIPA; 50 mM Tris-Hcl pH 7.5, 150 mM NaCl, 1 mM EDTA, 1% NP-40, 0.25% sodium deoxycholate at 90%, 1 mM Na3VO4, 1 *μ*g/mL leupeptin, 2 *μ*g/mL pepstatin, 1 *μ*g/mL aprotinin, and 0.1 mM PMSF; Sigma, Buchs, Switzerland). Muscle protein was quantified with a bicinchoninic acid kit (Pierce) against bovine serum albumin (BSA). Total protein was denatured in Laemmli buffer (50 mM Tris-HCL, pH 6.8, 10% glycerol, 2% SDS, 2%-mercaptoethanol, and 0.1% bromophenol blue) by heating for 5 min at 95°C. Equal amounts of total muscle protein per lane (i.e., 20 micrograms) were resolved via 7.5% sodium dodecyl sulphate-polyacrylamide gel electrophoresis (SDS-PAGE) using a Mini-Protean III system (Biorad). The loading was in a paired design with four pre-/post-ULLS samples from SOL or VL muscle of subjects per gel loaded in adjacent lanes. Proteins were blotted onto nitrocellulose membrane (Amersham) and blotting efficiency and equal loading were verified by Ponceau S staining. Membranes were subjected to immunodetection with specific first antibodies and horse radish peroxidase- (HRP-) coupled secondary antibodies. For the detection of FAK-related proteins, this involved the polyclonal C-terminal antibody from animal “Lulu” as published [38] and anti-rabbit HRP antibody (ICN Biomedicals GMBH, Germany). For vinculin, this included a described monoclonal antibody [39] and anti-mouse HRP antibody (ICN). For detection of the mitochondrial proteins SDHA, ATP5A1, UQCRC1, and NDUFA9, an antibody mix (Molecular Probes/Invitrogen Ltd, Paisley, UK) was used. UCP3 was detected using antibody #AB3046 (Millipore). For the detection of slow-type myosin heavy chain, MHCI, a monoclonal antibody (Sigma Chemicals, Buchs, Switzerland), and anti-mouse HRP antibody (ICN Biomedicals GMBH, Germany) were used.

Signal detection was carried out with enhanced chemiluminescence (Femto kit, Pierce) and quantified with a Chemidoc system running under Quantity One software (Bio-Rad, Life Science Research, Hercules, CA, USA). The signal intensity of the respective band was estimated with the “volume rectangular tool” and corrected versus the background of a band of equal height and size (area) in an empty sample lane. Background-corrected data were normalized to the mean values of the pre-ULLS samples for the respective gel; the values therefore reflect relative expression levels per total muscle protein. In addition, blots combining sample pairs from SOL and VL muscle were run on the same gel to compare and adjust relative protein content between the two muscle groups.

The content of FAK-pY397 per total protein was assessed as described [37]. In brief, the soluble fraction of 500 micrograms of total protein in 1 mL RIPA buffer was subjected to immunoprecipitation over night with 1 mg FAK-pY397-specific antibody (Invitrogen) and 50 microliters of a 10% slurry of protein A Sepharose (Sigma) at 4°C under continuous rotation using an Intellimixer (Progen Scientific). The precipitate of a 2-minute spin at 5000 g at 4°C was washed twice with 500-microliter RIPA, separated by 7.5% SDS-PAGE, western blotted onto nitrocellulose, and divided into two parts at the height of 85 kDa before being subjected to immunodetection with FAK-specific antibody as described above.

### 2.5. Proteasomal Activity

Homogenates were prepared from 20-micrometer cryosections in 0.1 M KH2PO4 buffer (pH 7.2) containing 2 mM EDTA on ice with the help of a polytron mixer (Kinematica, Switzerland). Protein concentration was quantified spectrophotometrically with the bicinchoninic acid protein assay kit (Perbio) and adjusted to 2 mg/mL. Samples were distributed in aliquots and stored at 60°C. The quantification of chymotrypsin-like enzyme activity of the 20S proteasome, representing the catalytic core of the proteasome, was based on the hydrolysis of a fluorogenic substrate essentially as described [36]. In brief, 20 micrograms of muscle extract was incubated in a final volume of 1 mL imidazole buffer (60 mM; pH 7.4). The reaction was started by the addition of 100 *μ*M of the fluorogenic substrate succinyl-leu-leu-val-tyr-7-amido-4-methylcoumarin (Bachem, I-1395). Substrate conversion was assessed fluorometrically (excitation at 380 nm and emission at 460 nm) on a SFM25 fluorometer (Kontron Instruments). Proteolytic activity was estimated based on a Michaelis Menten type model of catalytic activity where the conversion rate (i.e., the increase in fluorometric signal per time) is proportional to* V*max. Measures were repeated in minute intervals over the first 10 minutes of the reaction and the mean conversion rate calculated. Proteolytic activity (i.e.,* V*max) was then calculated versus standard curve of amido-4-methylcoumarin drawn from dilutions in the range of 0–150 pmol/mL.

### 2.6. Fibre-Type Percentage and Fibre Type Cross-Sectional Area Analysis

15-micrometer cryosections of VL and SOL muscle were incubated with a 1 : 400 dilution of mouse monoclonal antibody against fast myosin heavy chain (Clone MY-32, Sigma-Aldrich) in 0.3% BSA in phosphate buffered saline (PBS) and then reacted with a 1 : 2000 dilution of horseradish peroxidase-conjugated anti-mouse IgG (ICN). Immunoreactivity was detected with substrate solution, 3-amino-9-ethylcarbazole in dimethylformamide (Sigma Chemicals, Buchs, Switzerland). Nuclei were counterstained with hematoxylin and embedded in Aquatex (Merck, Germany). Sections were then processed to assess fibre-type composition and cross-sectional area of slow-type and fast fibres.

Myosin heavy chain-stained sections were recorded digitally at 10x magnification on Axioskop 2 microscope (Carl Zeiss Ltd, Welwyn Garden City, UK) that was operated with AxioVision software (Carl Zeiss Ltd, Welwyn Garden City, UK). Subsequently, one field from fast-type myosin heavy chain stained sections was assessed for cross-sectional area of stained (fast-type) and unstained (or slow-type) fibres against a scale by manually recording the periphery of each assessed fibre within the ARDOM software [39]. This differentiation was based on the observation that the large majority of muscle fibres in human* vastus lateralis* muscle are pure type I or type II fibres [39]. These numbers were used to calculate the percentage and mean cross-sectional area of slow- and fast-type muscle fibres. The area content of slow-type fibres was calculated using the formula [area content of slow-type fibre percentage of slow-type fibres × mean cross-sectional area of slow-type fibres/100%]. On average, 159 slow- and 36 fast-type muscle fibres were counted per muscle cross section of SOL and an average of 88 slow- and 87 fast-type muscle fibres were counted per muscle cross section of VL.

### 2.7. Reverse-Transcriptase Quantitative Polymerase Chain Reaction (RT-qPCR)

The levels of corresponding gene transcripts encoding the assessed mitochondrial proteins, FAK, and regulatory factors of proteasome degradation were assessed. RNA was isolated from muscle biopsies and subjected to RT-PCR as described [4]. Briefly, total RNA was extracted from muscle biopsies using TRIzol (Invitrogen Life Technologies Carlsbad, CA) according to manufacturer's protocol. Two micrograms of the RNA was synthesized into cDNA using reverse transcriptase (Superscript II RNase H, Invitrogen Life Technologies) and random primers (Roche Diagnostics) in a total volume of 20 *μ*L. Real-time PCR was performed on an ABI-PRISMA 7700 Sequence Detector System (Perkin-Elmer Applied Biosystems, Foster City, CA). For each reaction, 5 *μ*L of the diluted single-stranded cDNA was mixed with 12.5 *μ*L of the 2 X TaqMan PCR Mastermix, 1.25 *μ*L gene-specific primers/probe set, and 6.25 *μ*L sterile dH2O. The thermal cycling protocol was as follows: 2 min at 50°C and 10 min at 90°C followed by 40 cycles at 95°C for 15 s and 60°C for 1 min. Primer pairs and probes were supplied as Taqman Reagent kits from Applied Biosystems Inc. (Carlsbad, CA, USA) and used according to the manufacturer's instructions (UBB: Hs00430290_m1, UBC: Hs00824723_m1, PSMA2: Hs00746751_s1, SDHA: Hs00417200_m1, ATP5A1: Hs00900735_m1, UQCRC1: Hs00163415_m1, NDUFA9: Hs00245308_m1, and FAK (PTK2): Hs01056457_m1). GAPDH (4326317E) was selected as an endogenous control to correct for potential variations in RNA loading (Applied Biosystems Inc., Carlsbad, CA, USA). For all genes, samples were amplified simultaneously in duplicate in one assay run as described previously. The relative distribution of the transcripts of interest was measured for each individual; a cycle threshold (CT) value was obtained by subtracting the GAPDH CT values from the respective target CT values. The expression of each target was then evaluated using the 2^−ΔCT^ (ΔCT method).

### 2.8. Statistical Analysis

Post- versus prechanges of assessed parameters and interaction effects of “subjects” and “muscle” (SOL, VL) were assessed with a repeated ANOVA (Friedmann ANOVA). Effects were localized with the post hoc test of Fisher for least significant difference or Wilcoxon in dependency whether data were normally distributed (Statistica 9.1). Results were visualized as pre- and postvalues or as the fold difference between post- and prevalues. Effects with *P* values < 0.05 were considered significant and 0.05 ≤ *P* < 0.10 was called a trend. Linear relationships were assessed based on Pearson correlations between normalized values using Statistica 9.1. Correlation coefficients and *P* values were displayed as correlation matrices through the use of Cluster and Tree software (http://rana.lbl.gov/EisenSoftware.htm). Relationships were filtered to only consider those meeting a threshold of *r* ≥ 0.65 and *P* ≤ 0.10. The results were exported as tiff file into PowerPoint (MS-Office). Eight pre-/postsample pairs were assessed for all the studied proteins, except for mitochondrial proteins where only seven sample pairs were analysed for VL muscle.

## 3. Results

### 3.1. Muscle Fibre Types with Early Unloading

**Figure 1:**
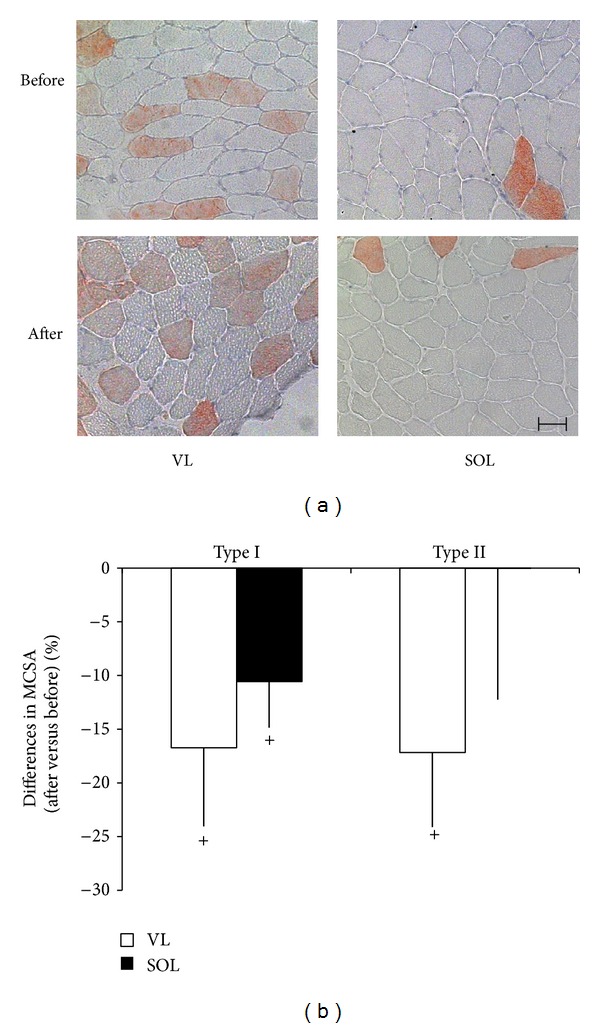
Fibre types with unloading. (a) Microscopic pictures of muscle cross sections of SOL and VL before and after ULLS after staining for type II myosin type heavy chain (orange). Nuclei appear in blue. The bar indicates 50 micrometers. (b) Mean ± SE of changes in MCSA for type I and type II muscle fibres in VL and SOL muscle within 3 days of ULLS. + denotes 0.05 ≤ *P* < 0.10 (two-tailed Fisher test).

**Figure 2:**
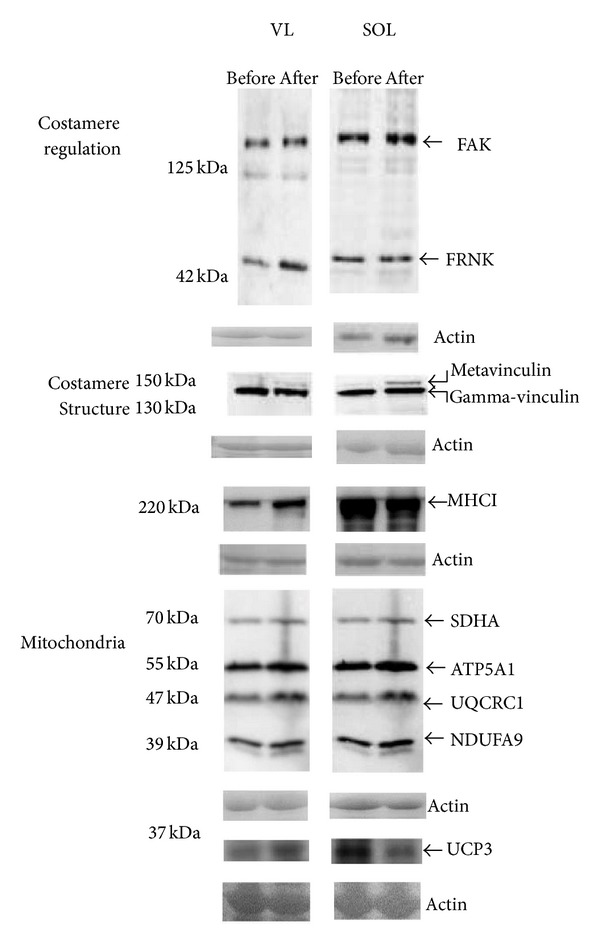
Protein detection in SOL and VL muscle. Representative immunoblots visualizing the detected proteins in SOL and VL before and after 3 days of ULLS. Arrows indicate the protein bands of interest. Below each blot, a loading control visualizes the actin band of the respective samples on the Ponceau S stained membrane. The position of molecular weight markers is shown to the left.

**Figure 3:**
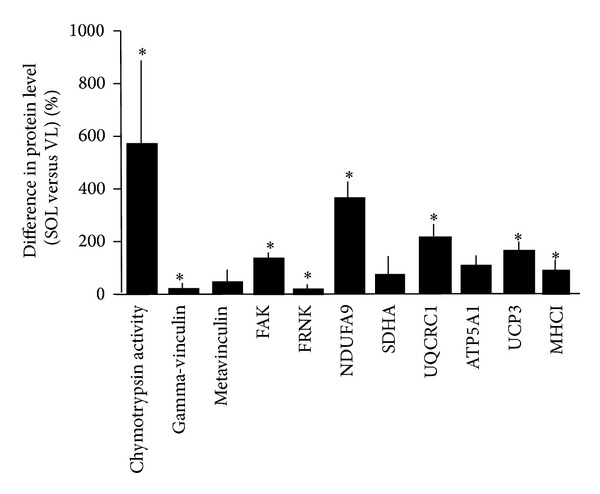
Differences in protein expression in the studied muscles. Mean and standard error of the percentage difference between SOL and VL muscle for the levels of the assessed proteins. Asterisk denotes significant difference between the SOL and VL muscle (*P* < 0.05, repeated ANOVA with post hoc test of Fisher).

Fibre-type distribution differed significantly between the VL and SOL muscles. Before ULLS, the SOL had 32% more type I fibres and a larger MCSA of type II fibres than did the VL muscle (Table 1, Figure 1).  Figure 2 shows representative immunoblots of the detected mitochondrial, costameric, and sarcomeric (MHCI) proteins in both muscles studied. The levels of MHCI, three mitochondrial proteins (NDUFA9, UQCRC1, and UCP3), the costamere-associated proteins FAK and FRNK, and chymotrypsin activity were higher in the SOL compared to the respective VL muscle (Figure 3).

Three days of ULLS caused trends for a reduction in MCSA of type I and type II, muscle fibres in the VL muscle (*P* = 0.076 and 0.054, resp.), and type I muscle fibres in the SOL muscle (*P* = 0.051) (Table 1(a); Figure 1(b)). Changes in MCSA of type I and type II muscle fibres were highly correlated in the VL muscle (*r* = 0.98), but they were not affected by fibre type in VL (*P* = 0.789) and SOL muscle (*P* = 0.340), respectively. Fibre-type distribution was not affected by three days of ULLS (Table 1(b)).

### 3.2. Effects of Unloading on Protein Expression

**Figure 4:**
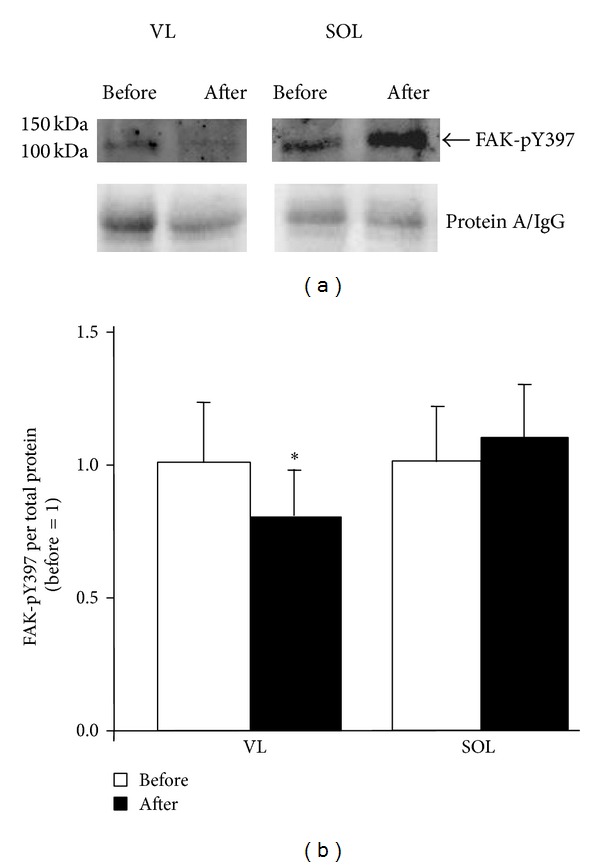
FAK-pY397 content in VL and SOL muscle before and after ULLS. (a) Top, example of immune-precipitated FAK-pY397 from total homogenate of VL and SOL muscle before and after ULLS for two subjects. The position of FAK-pY3967 is indicated. Bottom, the amount of immunoglobulin/protein is shown as a loading control in the panel below. (b) Bar graph showing mean and standard error of FAK-pY397 levels in SOL and VL muscle before and after 3 days of ULLS. ∗ denotes *P* < 0.05.

**Figure 5:**
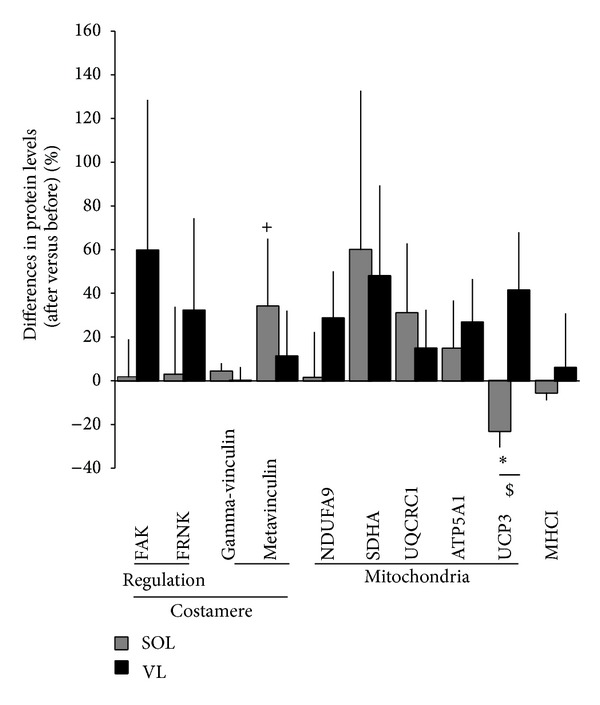
Changes in protein content with unloading. Mean and standard error of protein level changes within 3 days of ULLS in VL and SOL muscle. Asterisk and cross indicate those gene ontologies and proteins which were significantly or as a trend affected by ULLS. ∗ and + denote *P* < 0.05 and 0.05 ≤ *P* < 0.10. $ indicates a significant interaction effect (*P* < 0.05, repeated ANOVA).

**Figure 6:**
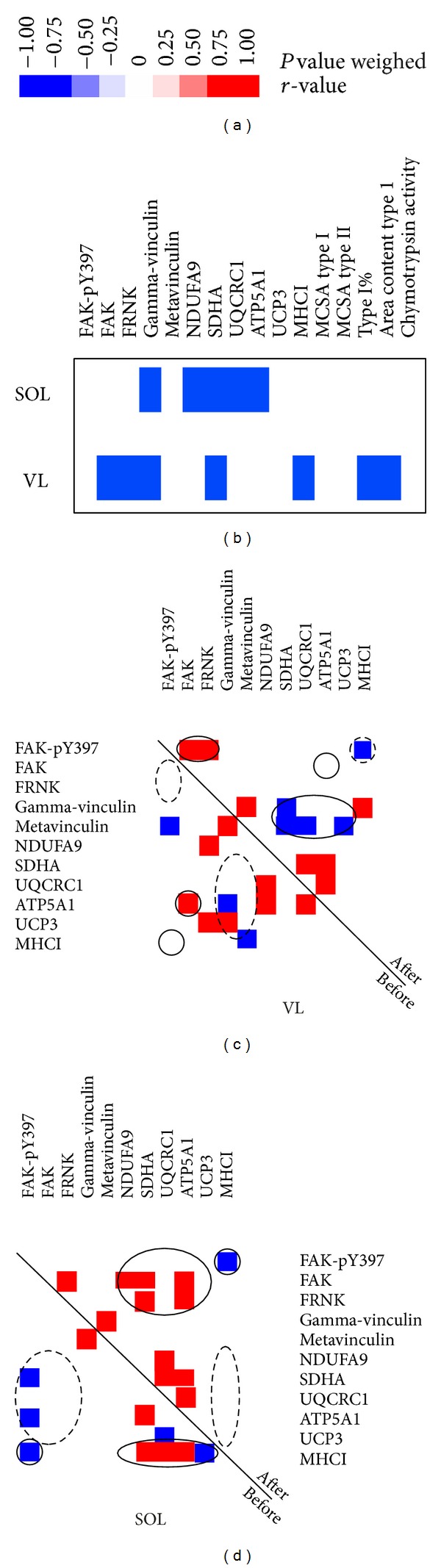
Correspondence in protein level alterations with unloading. ((a), (b)) Heat map visualizing the correlation between fold-changes in the content of assessed muscle parameters and values before ULLS (b). *P* value weighed *r*-values are given in color-coded boxes (a). ((c), (d)) Correlation matrices visualizing the changes in linear relationships in the VL (c) and SOL muscles (d) within 3 days of ULLS. Only relationships with an *r*-value *r* ≥ 0.65 and *P* ≤ 0.10 are considered for the display. Transitions can be visualized by comparing relationships before versus after ULS along the symmetry axes. Circles denote relationships of specific interest.

A tendency toward decreased FAK mRNA content was observed after ULLS in the VL (Table 2; *P* = 0.075). Neither the VL nor the SOL showed altered FAK protein levels after ULLS. FAK-pY397 content relative to total protein content was reduced by 20% in the VL but not in the SOL (Figure 4). There was a main effect of “subject” and “muscle” on the change in protein levels from before to after unloading (*P* = 0.001). Metavinculin content demonstrated a trend for an increase after 3 days of ULLS in the SOL (*P* = 0.058) but not in the VL muscle (*P* = 0.460; Figure 5). The content of the four mitochondrial proteins assessed, which are involved in electron transport and coupled ATP synthesis, was unaltered in both of the VL and SOL after unloading. The content of the mitochondrial protein UCP3 was reduced in the SOL muscle, which differed from the response observed in the VL muscle (*P* = 0.012). The fold changes of several proteins from before to after the 3-day ULLS were correlated inversely with their respective values before the intervention in both of the VL and SOL muscle (Figure 6(b)). The expression of one corresponding transcript, ATP5A1, was reduced (Table 2).

### 3.3. Proteasomal Gene Expression and Activity

The number of transcripts for the proteasomal subunit PSMA2 was increased in both muscles after ULLS, whereas that of UBC was increased in the SOL exclusively. Trends toward increases in both UBB and UBS were observed in the VL (*P* = 0.058 and 0.093, resp.). The activity of chymotrypsin, which is a main contributor to proteasomal activity, was unaltered in both of the VL (*P* = 0.565) and SOL (*P* = 0.423) after ULLS.

### 3.4. Relationships between Muscle Fibre MCSA and Chymotrypsin Activity

There was a significant interaction effect between “subject” and “muscle” regarding changes in the MCSA of muscle fibres from before to after ULLS (*P* = 0.014). This finding suggests interindividual and muscle-specific differences in response to reduced weight bearing. The fold changes in chymotrypsin activity in the VL muscle were correlated negatively with the changes in the MCSA of type II fibres (*r* = −0.72, *P* = 0.049).

### 3.5. Deregulated Coordination of Expression and Linear Relationships with the Slow Oxidative Muscle Phenotype

Correlation matrices visualized changes in the linear relationships observed in the muscles from before to after ULLS. Transitions were visualized by comparing the relationships from before to after unloading along the symmetry axes (Figures 6(c) and 6(d)). There was a positive correlation between respiratory-chain constituents and MHCI content in the SOL before, but not after, ULLS. In the VL, a negative correlation between FAK-pY397 and MHCI was established after unloading, which was the default situation in the SOL muscle.

## 4. Discussion

Reduced weight bearing induces muscle loss mainly by decreasing anabolic drive because of decreased protein synthesis and increased proteolysis [5, 15, 16]. The quantitative importance of modified gene expression for the early structural changes in human muscle fibres that lead to atrophy is not fully understood [10, 40]. Previous investigations of transcript expression in rodent antigravity muscles in the presence of unloading [19, 41] indicate that the regulation of slow oxidative gene expression in the soleus muscle is FAK dependent [20]. The present study demonstrated a muscle-specific transition in the correlation pattern of factors that reflect the slow oxidative phenotype (Figure 6) and a reduced activity status of FAK and reduced content of UCP3, which decouples electron transport from ATP synthesis [42] (Figures 4 and 5). The current investigation is the first to corroborate the notion of early molecular changes at the protein level after discontinued weight bearing in human antigravity muscles.

Consistent with the previously reported time pattern of muscle loss after reduced weight bearing [2, 6, 7, 43], our data showed a trend for a reduction in the MCSA of muscle fibres after 3 days of ULLS (Figure 1(b)). However, a multifactorial analysis of variance identified an interaction effect of “muscle” and “subject” on changes in muscle fibre cross-sectional area after ULLS. This result suggests the presence of interindividual differences and muscle-specific reactions induced by reduced weight bearing. This notion was supported by the significant intersubject variability observed in the changes in protein levels in response to ULLS (data not shown). A correlation analysis revealed that unloading-induced changes in costameric, mitochondrial, and MHCI proteins in general could be explained by subject variability at the baseline (Figure 6(b)). We believe that this statistical approach allows the identification of the overall deregulation of protein expression in response to short-term unloading, before this is detected at the single-protein level. Consistent with this notion, we observed alterations in the correlations between the levels of mitochondrial proteins, MHCI, and components and regulators of their costameric anchor in the sarcolemma [24] with unloading (Figure 6). For instance, there was a loss of correlation between the levels of MHCI and the mitochondrial proteins SDHA, UQCRC1, ATP5A1, and UCP3 in the SOL muscle after ULLS. This modification was associated with a trend for increased metavinculin content in the SOL muscle. The latter change is consistent with the effects of 84 days of bed-rest [30]. Altogether, the present observations suggest an early transit of muscle fibres in a remodelling phase with reduced load bearing, which later involves shifts in myosin isoform expression [10, 30]. Differences in the pattern of correlations with unloading and the specific reduction in UCP3 content in SOL compared with the VL muscle (Figure 5) indicate that these reactions after 3 days of ULLS are muscle specific.

Changes in the metabolic profile and contractile composition of muscle fibres have been reported after 2 weeks of unloading in both rats and humans [8, 44]. Alterations of mitochondrial proteins and MHCI appear to be regulated in a load-dependent manner via the phosphorylation of FAK at FAK-pY397 [20, 45]. Hence, FAK is enabled to develop its full catalytic activity and phosphorylate auxiliary sites (e.g., tyrosine 576/577) and interact with further binding partners, thereby possibly affecting the assembly and turnover of focal adhesions [33], ribosome biogenesis, and sarcomerogenesis [20, 37]. In support of such a mechanism in human muscle, we found reduced FAK-pY397 content in the VL after ULLS. This was associated with the disappearance of the correlation between FAK-pY397 and the FAK-regulated costamere component, metavinculin (Figure 6; [27]). Concomitantly, the correlation between meta- and gamma-vinculin and mitochondrial proteins (SDHA, UQCRC1, ATP5A1, and UCP3) in the VL muscle was lost after unloading (Figure 6). This transition corresponds to the greater molecular alterations that accompany 3 days of ULLS [4].

Three days of unloading have been shown to increase the proteolysis of contractile proteins in the VL muscle [5]. The proteasomal machinery is held as a major contributor to the sequenced process that degrades myofibrils [36, 46]. In support of this role, our correlation analysis revealed the presence of a linear relationship between fold changes in the activity of chymotrypsin that constitutes a main component of the proteasome and the MCSA of type II fibres in the VL muscle after 3 days of ULLS (*r* = −0.71). Our finding is compatible with the previously identified upregulation of the mRNAs for the ubiquitin ligases atrogin-1 and MuRF-1 (which direct the activity of the proteasomal pathway) in the VL compared with the SOL [4], suggesting an enhanced capacity for proteasomal degradation of myofibrillar protein in VL muscle with unloading. Conversely, the increase in the* UBC* and* PSMA2* gene transcripts in SOL and VL muscle (Table 2) suggests that expressional regulation towards an increased capacity for proteasomal degradation takes place in both muscles after only 3 days of ULLS. In support of this view, we observe a selective reduction in the protein content of the proteasome target UCP3 [47] in SOL muscle after ULLS (Figure 5) and find that the post-/predifferences in UCP3 protein levels correlate with those of chymotrypsin activity (*r* = −0.81; *P* = 0.093). Correlations analysis identifies that the muscle phenotype contributes to baseline differences in chymotrypsin activity at baseline (Figure 3). This suggests a contribution for chymotrypsin-mediated proteolysis in setting variability of the transformation of oxidative characteristics after unloading, as well as mitochondrial protein turnover, in the two muscles being studied.

Our report is the first to document the regulation of UCP3 in the presence of unloading in humans. In addition to the observed changes in correlations between proteins, the downregulation of the mitochondrial transcript ATP5A1 was observed in both of the VL and SOL muscles (Table 2). These observations support the earlier contention of compromised mitochondrial function [10–13] with unloading. Interestingly, the mitochondrial protein UCP3, which is involved in the dissipation of mitochondrial energy, was negatively correlated to MHCI content at baseline (Figure 6(d)) and specifically reduced in the slow oxidative SOL muscle. This suggests muscle-specific alterations in energy homoeostasis during early unloading.

One of the limitations of our approach was that, to exclude effects from prior tissue sampling, pre- and postbiopsy samples were collected from different legs. It is not possible to exclude differences at the baseline in costamere components between the two legs, which might have influenced our results. However, our observations of stable FAK protein content after 3 days of ULLS, in combination with a reduced Y397 phosphorylation of FAK, do not support the possibility that random variation obscured the current findings, because FAK is expected to be affected by prolonged alterations in muscle use and loading [22, 27, 39]. Moreover, the similar fibre percentage observed in the two legs pre- versus post-ULLS does not support the presence of systematic effects induced by the biopsy protocol, because of the known association between FAK content and fibre percentage. At last, we acknowledge that mechanistic relationships cannot be directly assessed in our observational human investigation. Thus, we assessed whether linear interrelationships (i.e., correlation) exist for selected molecular muscle parameters which we have identified to be regulated in a load-dependent manner in rat muscle by FAK protein and FAK-pY397 content [20, 37]. In spite of the relatively low number of subjects, we identify a number of significant correlations between proteins that set the slow oxidative gene program and costamere components that are consistent with the conclusions from our mechanistic intervention [20, 37] and which resemble earlier notions of correlative relationships between the levels of metabolic proteins in a given muscle type [48].

## 5. Conclusions

Evidence for muscle remodelling can be detected at the protein level in human antigravity muscles within 3 days of unloading. The reduced FAK-pY397 content observed in the VL indicates the modification of costameres in the early adaptation to unloading, as observed previously in the rat. Baseline levels and proteolytic activation influence the individual response of sarcomeric and mitochondrial proteins. Although the current findings cannot explain the more rapid and substantial atrophy reported previously in the SOL, differences in the early phase response were observed between the VL and SOL muscles. No change in FAK-pY397 content was observed in the SOL muscle; however, this muscle showed a reduced content of uncoupling protein 3 indicating that the first modifications in muscle make-up in the presence of unilateral limb unloading are muscle specific and initiate within 3 days of unloading.

## Figures and Tables

**(a):** 

MCSA type I	[*μ*m^2^]
VL	5275 ± 442
SOL	6330 ± 479
*P* value	0.102

MCSA type II	[*μ*m^2^]

VL	6225 ± 292
SOL	7353 ± 382
*P* value	0.026

**(b):** 

Type II [%]	Before	After	*P* value (before versus after)
VL	49.3 ± 7.1	57.5 ± 5.7	0.347
SOL	14.2 ± 4.2	17.1 ± 3.9	0.364

*P* value (VL versus SOL) is 0.001.

**Table 2:** Altered transcript expression with muscle unloading. Mean percentage of post- versus prechanges in GAPDH-standardized mRNA levels in VL and SOL muscle within 3 days of ULLS. *P* values of Wilcoxon tests are given with values and changes with *P* ≤ 0.05 are indicated in bold.

	VL	*P* value	SOL	*P* value
UBB	9.4 ± 11.6%	0.058	16.6 ± 26.5%	0.379
UBC	36.1 ± 29.0%	0.093	**52.0 ± 22.0%**	0.011
PSMA2	**35.1 ± 24.5%**	0.046	**54.9 ± 32.0%**	0.010

SDHA	0.2 ± 10.0%	0.465	−17.3 ± 9.2%	0.359
ATP5A1	**−5.7 ± 6.5%**	0.028	**−25.3 ± 10.3%**	0.006
UQCRC1	6.8 ± 14.5%	0.345	3.6 ± 19.2%	0.534
NDUFA9	17.3 ± 11.2%	0.091	30.1 ± 44.7%	0.529

FAK	17.6 ± 22.9%	0.075	19.1 ± 27.2%	0.158
